# CAG regimen for refractory or relapsed adult T‐cell acute lymphoblastic leukemia: A retrospective, multicenter, cohort study

**DOI:** 10.1002/cam4.3079

**Published:** 2020-06-03

**Authors:** Jie‐Jing Qian, Xiaoxia Hu, Ying Wang, Yi Zhang, Juan Du, Min Yang, Hongyan Tong, Wen‐Bin Qian, Juying Wei, Wenjun Yu, Yin‐Jun Lou, Liping Mao, Hai Tao Meng, Liang‐Shun You, Libing Wang, Xia Li, Xin Huang, Li‐Hong Cao, Jian‐Zhi Zhao, Xiao Yan Yan, Yu‐Bao Chen, Yu Chen, Su‐Jiang Zhang, Jie Jin, Jiong Hu, Hong‐Hu Zhu

**Affiliations:** ^1^ Department of Hematology the First Affiliated Hospital College of Medicine Zhejiang University Zhejiang China; ^2^ Institute of Hematology Zhejiang University Zhejiang China; ^3^ Zhejiang Province Key Laboratory of Hematology Oncology Diagnosis and Treatment Hangzhou China; ^4^ Department of Hematology Institute of Hematology Changhai Hospital Shanghai China; ^5^ Department of Hematology Shanghai Jiaotong University School of Medicine Affiliated Ruijin Hospital North Shanghai China; ^6^ Department of Hematology Shanghai Changzheng Hospital The Second Military Medical University Shanghai China; ^7^ Department of Hematology Shulan (Hangzhou) Hospital Hangzhou China; ^8^ Department of hematology Shaoxing Central Hospital Shaoxing China; ^9^ Department of Biostatistics Peking University Clinical Research Institute Beijing China; ^10^ Shanghai Institute of Hematology Department of Hematology, Blood and Marrow Transplantation Center Collaborative Innovation Center of Hematology RuiJin Hospital Shanghai Jiao Tong University School of Medicine Shanghai China

**Keywords:** refractory or relapse, stem cell transplantation, T‐cell acute lymphoblastic leukemia

## Abstract

Adult patients with relapsed or refractory T‐cell acute lymphoblastic leukemia (R/R‐T‐ALL) have extremely poor prognosis, representing an urgent unmet medical need. Finding an optimal salvage regimen to bridge transplantation is a priority. The CAG (cytarabine, aclarubicin, and G‐CSF) regimen was initially used by one group in China, showing unexpectedly promising results in 11 R/R‐T‐ALL patients. Here, we report the multicenter results of 41 patients who received the CAG regimen as salvage therapy. After one cycle of the CAG regimen, complete remission and partial remission were achieved in 33 (80.5%) and two (4.9%) patients, respectively. Failure to respond was observed in six patients (14.6%). Early T‐cell precursor (ETP) (n = 26) and non‐ETP (n = 15) patients had a similar CR rate (80.8% vs 80.0%, *P* = .95). Among 41 patients, allo‐HSCT was successfully performed in 27 (66%) patients (22 in CR and 5 in non‐CR). With a median follow‐up time of 12 months, the estimated 2‐year overall survival and event‐free survival were 68.8% (95% CI, 47.3%‐83.0%) and 56.5% (95% CI, 37.1%‐71.9%), respectively. The CAG regimen was well‐tolerated, and no early death occurred. Our multicenter results show that the CAG regimen is highly effective and safe, representing a novel choice for adult patients with R/R‐T‐ALL and providing a better bridge to transplantation.

## Graphical abstract

Adult patients with relapsed or refractory T‐cell acute lymphoblastic leukemia (R/R‐T‐ALL) have limited therapeutic options and extremely poor prognosis (CR rate <50% and long‐term survival <20%), which represents an urgent and unmet medical need. Finding an optimal salvage regimen that has a good response and is well‐tolerated to bridge to transplantation is a priority.

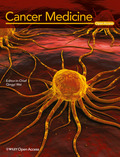

## 1 INTRODUCTION

Adult T‐cell acute lymphoblastic leukemia (T‐ALL) accounts for approximately 25% of adult ALL cases and has inferior outcomes.^1^, ^2^, ^3^ Although the outcomes have improved slowly in recent years for newly diagnosed patients, refractory or relapsed T‐ALL (R/R‐T‐ALL) patients have extremely poor outcomes, with <10% of patients surviving long term.^4^, ^5^, ^6^, ^7^, ^8^, ^9^, ^10^, ^11^, ^12^, ^13^, ^14^, ^15^, ^16^ The only curable treatment is initiating a salvage regimen to achieve complete remission (CR) and then rapidly performing allogeneic hematopoietic stem cell transplantation (allo‐HSCT).^9^ Therefore, selecting an effective salvage regimen is vital for R/R‐T‐ALL.

Until now, there has been no consensus about optimal salvage treatments for R/R‐T‐ALL, representing an urgent, unmet medical need.^4^, ^5^, ^6^ Conventional chemotherapy options, such as FLAG (fludarabine, cytarabine, and granulocyte colony‐stimulating factor), with or without idarubicin and clofarabine in combination with etoposide and cyclophosphamide, or even the only T‐ALL target drug (nelarabine), have been used, but the overall response rates are only 30%‐50%.^8^, ^14^, ^17^, ^18^ Therefore, a more effective and safer salvage regimen for R/R‐T‐ALL is highly awaited.

The CAG regimen [cytarabine (C), aclarubicin (A), and granulocyte colony‐stimulating factor (G)], which is conventionally used in China in acute myeloid leukemia (AML),^19^ showed unexpectedly promising preliminary results for the treatment of R/R‐T‐ALL in the pilot study performed by the Xue group.^20^, ^21^, ^22^ In fact, 10 of 11 patients with R/R‐T‐ALL achieved CR.^21^ The limited same size prompted us to further extend the study to validate these results of the CAG regimen for R/R‐T‐ALL in a multicenter setting. Here, we report the results of a multicenter study by the Chinese Leukemia Cooperation Group (CLCG) to evaluate the efficacy and safety of the CAG regimen for the treatment of adult R/R‐T‐ALL.

## 2 METHODS

### 2.1 Study design and data collection

This was a multicenter (six centers), retrospective analysis. The presence of R/R‐T‐ALL was confirmed. Eligibility criteria were age 16 years or older, and CAG regimen as salvage therapy from September 2012 to June 2019. The study was conducted in accordance with the Declaration of Helsinki, and approval was granted by the relevant ethics committees.

The CAG regimen (cytarabine, 10 mg/m^2^ subcutaneously every 12 h on days 1‐14 or 20 mg/m^2^ subcutaneously every 12 h on days 1‐7; aclarubicin, 20 mg intravenously daily on days 1‐4; and granulocyte colony‐stimulating factor (G‐CSF), 200 mg/m^2^ per day subcutaneously from day 1 until neutrophil recovery) was administered. After achieving CR, patients received the same CAG regimen or other regimens as subsequent consolidation and then allo‐HSCT as soon as possible if a suitable donor was available.

Early T‐cell precursor (ETP) ALL (ETP‐ALL) was defined as leukemia characterized by the absence (<5% positive cells) of CD1a and CD8 expression, weak CD5 expression (<75% positive cells), and expression (>25% positive cells) of at least one or more of the following myeloid or stem cell markers: CD117, CD34, human leukocyte antigen (HLA)‐DR, CD13, CD33, CD11b, and CD65.^23^, ^24^

### 2.2 Statistical analysis

The measured outcomes were CR, partial response (PR), OS, and event‐free survival (EFS). CR was defined as <5% blasts in the bone marrow (BM) with normalization of peripheral blood counts (ANC ≥ 1×10^9^/L and platelet count ≥100×10^9^/L) and complete resolution of extramedullary disease. PR was defined similarly, except for the presence of 6 to 25% BM blasts.^15^, ^25^, ^26^, ^27^

Relapse was defined as the reappearance of blasts in the blood or in the bone marrow (>5%) or in any other extramedullary site after a CR.^25^ Refractory disease was defined as the failure to achieve CR at the end of induction therapy. 25 OS was calculated as the interval between the diagnosis of refractory disease or relapse and death from any cause. EFS was defined as the time between the diagnosis of refractory disease or relapse and the day of the first adverse event (salvage failure, further relapse, or death from any cause). Survival probabilities were analyzed by the Kaplan‐Meier method. Outcomes were compared between the different subgroups by the log‐rank test. The independence of the categorical parameters was calculated using either a χ^2^ test or Fisher's exact test. The distribution of the continuous variables was calculated using a Mann‐Whitney U test. *P* < .05 were considered significant.

## 3 RESULTS

**FIGURE 1:**
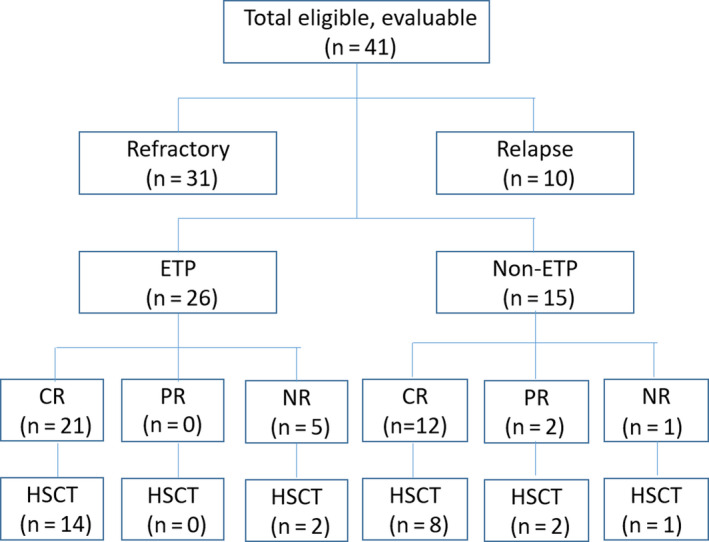
Overview of patients included in the analysis. CR, complete remission; ETP, Early T‐cell precursor; HSCT, hematopoietic stem cell transplantation; PR, partial remission; NR, no response

The characteristics of all 41 patients are shown in Figure 1 and Table 1. There were 28 males and 13 females, and the median age was 31 years (range, 15‐78 years). The numbers of patients with ETP‐ALL and non‐ETP‐ALL were 26 (63.4%) and 15 (36.6%), respectively. There were 31 (75.6%) primary refractory patients who had undergone 1‐3 prior salvage chemotherapy cycles including the VDLP, VDCP, or hyper‐cyclophosphamide, vincristine, doxorubicin and dexamethasone (CVAD) regimens, and 10 relapsed patients (24.4%) (3 of 10 patients had undergone 1‐2 prior salvage chemotherapy regimens) with a median relapse time of 14.5 months (4‐48 months) (Table 1). The last follow‐up time was 1 July 2019, and the median follow‐up time was 12 months (range, 1‐63 months). There were 30 patients alive at the last contact, and 11 patients died of the disease.

As shown in Table 1, after one cycle of the CAG regimen among 41 patients, complete remission and partial remission were achieved in 33 (80.5%) and two (4.9%) patients, respectively. Failure to respond was observed in six patients (14.6%). ETP (n = 26) and non‐ETP (n = 15) patients had a similar CR rate (80.8% vs 80.0%, *P* = .95). No patients experienced an early death within 1 month after the initiation of the CAG regimen. Among 41 patients, allo‐HSCT was successfully performed in 27 (66%) patients (22 in CR and 5 in PR/NR), and 24 patients receiving allo‐HSCT were still alive at the last follow‐up. Thirty‐three patients achieved CR after CAG with the intent of being transplanted, and 22 patients actually reached transplantation. Among 22 patients who received allo‐HSCT after achieving CR from CAG, 10 patients directly bridged to allo‐HSCT without consolidation, eight patients repeated the CAG regimen, and four patients received hyper‐CVAD as consolidation before allo‐HSCT.

**FIGURE 2:**
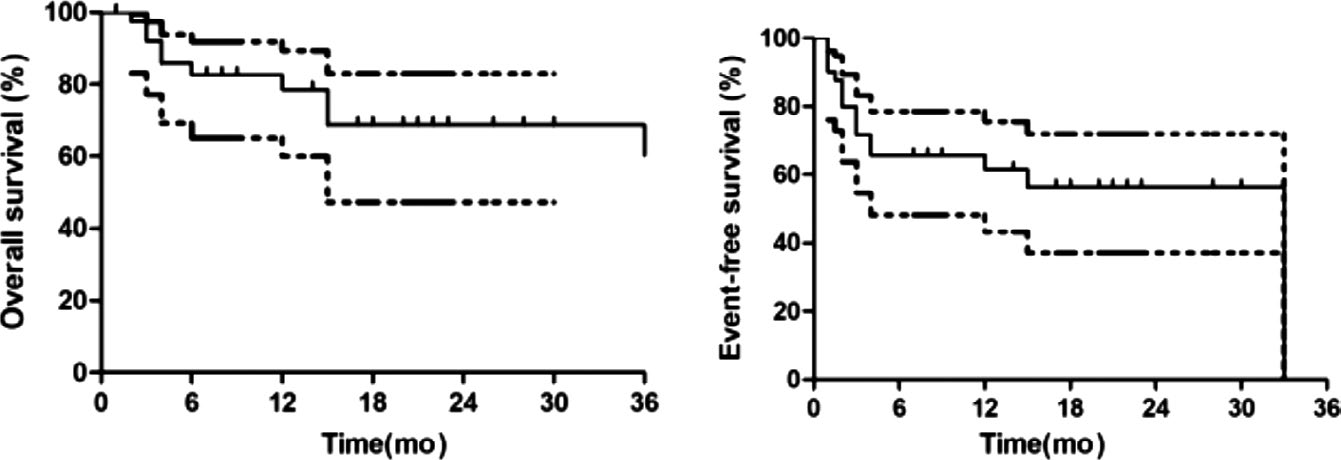
The overall survival and event‐free survival of all the relapse or refractory T‐ALL patients

**FIGURE 3:**
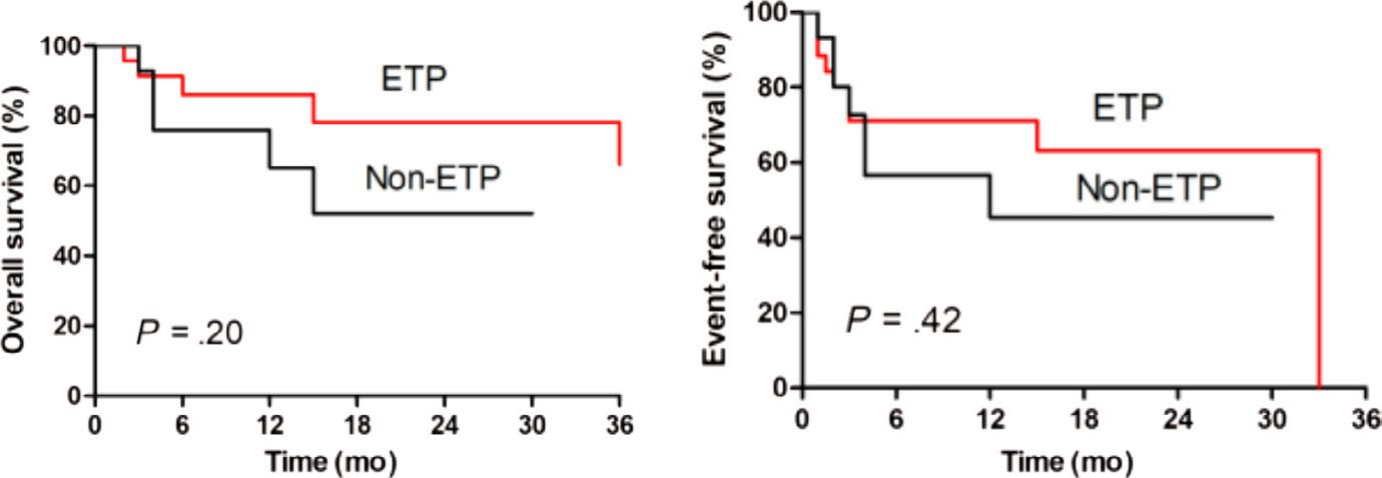
The overall survival and event‐free survival of T‐ALL patient with early T‐cell precursors (ETP) or non‐ETP salvaged with CAG regimen

In a total of 41 patients, the estimated 2‐year OS was 68.8% (95% CI, 47.3%‐83.0%), and the EFS was 56.5% (95% CI, 37.1%‐71.9%) (Figure 2). ETP and non‐ETP patients had similar OS (78.3% vs 52.1%, respectively, *P* = .20) and EFS (63.2% vs 45.3%, respectively, *P* = .42) values (Figure 3).

Adverse events (AEs) were graded according to the National Cancer Institute Common Terminology Criteria for Adverse Events, version 4.03. As shown in Table 2, grade 3 to 4 neutropenia and thrombocytopenia occurred in 30 (73%) and 32 (78%) patients, respectively. The median duration of neutropenia during the first cycle of CAG therapy was 10 days (range 6‐28 days). The most common nonhematologic AE was infection (grade 3‐4, 58%). The infections that occurred during CAG chemotherapy were in the lung (55.6%), gastrointestinal tract (14.8%), skin (11.1%), bloodstream (11.1%), and unknown locations (7.4%). No cases of grade 3 to 4 nausea, vomiting, diarrhea, thrombosis/embolism, cardiac events, raised creatinine, or nervous system symptoms occurred. No early deaths occurred during the CAG regimen.

**FIGURE 4:**
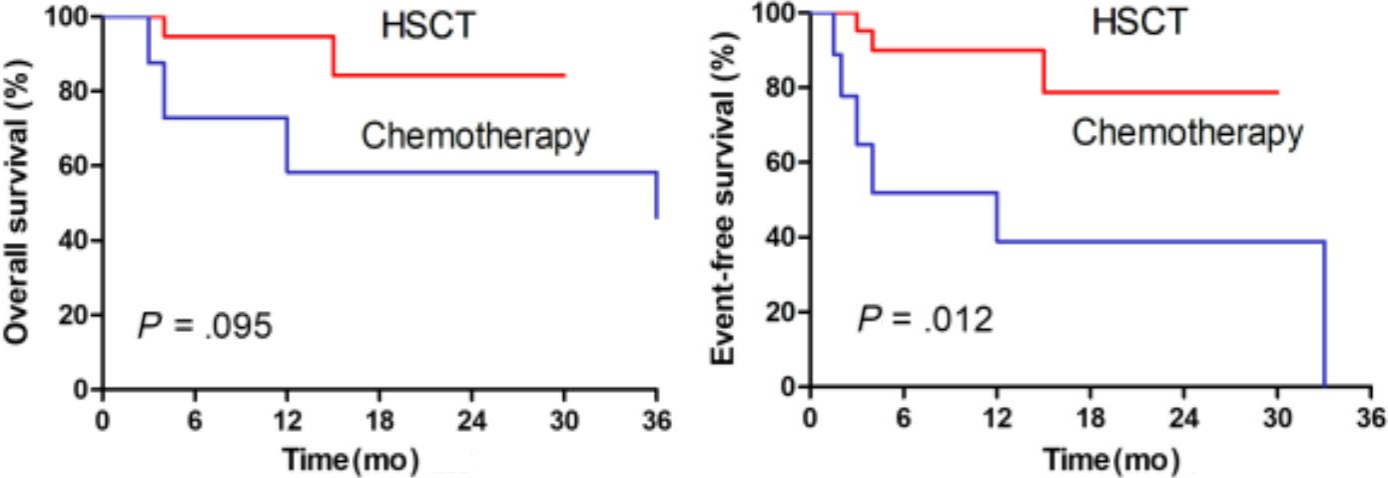
The overall survival and event‐free survival of R/R‐T‐ALL patients who received allo‐HSCT or chemotherapy after achieving CR with CAG regimen

Among the 33 patients achieving CR after the CAG regimen, 22 patients successfully bridged to allo‐HSCT, and 11 patients received chemotherapy‐based postremission treatments. In the patients who successfully bridged to allo‐HSCT and the patients who received chemotherapy‐based postremission treatments, the estimated 2‐year OS rates were 84.2% (95% CI, 47.0%‐96.2%) and 58.3% (95% CI, 18.0%‐84.4%) (*P* = .095), respectively; the estimated 2‐year EFS were 78.7% (95% CI, 43.2%‐93.4%) and 58.3% (95% CI, 9.3%‐68.8%) (*P* = .012), respectively (Figure 4).

## 4 DISCUSSION

Adult patients with R/R‐T‐ALL have poor outcomes. As such, a novel therapy is urgently needed.^4^, ^5^, ^6^, ^7^, ^8^, ^9^, ^10^, ^11^, ^12^, ^13^, ^14^, ^15^, ^16^ Our multicenter results show that the CAG regimen is associated with a high CR rate of 80.5% and is well‐tolerated, enabling most patients to bridge to allo‐HSCT. Thus, this regimen represents a novel option for adult R/R‐T‐ALL patients. These promising results prompted a prospective multicenter trial that was initiated in June 2019 in China (Chinese Clinical Trial Registry number, ChiCTR1900023581).

The most interesting finding of this study, which included a relatively large sample size of patients from multiple centers in China, was the very high ORR rate (85.4%) associated with the use of the CAG regimen for R/R‐T‐ALL patients, confirming the previous results found in studies with smaller sample sizes from single centers.^20^, ^21^, ^22^ The CAG regimen is commonly and exclusively used for R/R‐AML or elderly AML patients in Asia because aclarubicin has not been launched in America and Europe. Empirically, one group in China found an unexpectedly promising primary result in which all six R/R‐T‐ALL patients diagnosed from April 2004 to July 2006 achieved CR.^20^ Subsequently, the same group extended the treatment to another five patients, four of whom achieved CR.^21^ Commonly used salvage regimens for R/R‐T‐ALL include FLAG‐based or hyper‐CVAD‐based regimens, which have CR rates varying from 30% to 50%.^4^, ^5^, ^6^, ^8^, ^14^, ^17^, ^18^ The only single‐target drug for T‐ALL is nelarabine, which has a CR rate varying from 31% to 36% as salvage monotherapy.^8^, ^10^, ^14^ A recent study from the Children's Oncology Group showed that the proteasome inhibitor bortezomib plus chemotherapy could increase the CR rate to 68% among 22 patients with once‐relapsed T‐ALL.^16^ According to the reported CR rates listed in Table 3, the CAG regimen seems to be a good salvage regimen for R/R‐T‐ALL, but the results should be interpreted cautiously for different eligible populations.

**TABLE 3:** Salvage regimens for refractory or relapsed T‐ALL

Reference	Study resource	Children/adult	Relapse/refractory	No. of T‐ALL patients	Reinduction regimen	CR, %	ED, %	Bridge HSCT, %	EFS, %	OS, %
Rivera 2005^7^	St. Jude Children's Research Hospital	Children	First relapse	20	Chemotherapy	60		15	5.0 (5y)	5.0 (5y)
Berg 2005^8^	COG study	Children	Refractory/relapse	106	Nelarabine	25.7	0.7			
Fielding 2007^9^	MRC UKALL12/ECOG 2993 study	Adult	First relapse	92	Chemotherapy					5.0 (4y)
DeAngelo 2007^10^	CALGB‐19801 study	Adult	Refractory/relapse	26	Nelarabine	31	0	26.9		28(1y)
Raetz 2008^11^	COG‐AALL01P2 study	Children	First relapse	7	Chemotherapy	28.8			0(5y)	0(5y)
Reismüller 2009^12^	Austria‐BFM‐study	Children	First relapse	28	Chemotherapy				21(10y)	21(10y)
Marks 2009^13^	UKALL XII/ECOG 2993	Adult	First relapse	123	Chemotherapy			22		6.5(5y)
Gökbuget 2011^14^	German Multicenter Study Group	Adult	Refractory/relapse	126	Nelarabine	36	0.8	28.6		11(6y)
O’Brien 2013^15^	Multi‐Centre of USA and Cananda	Adult	Refractory/relapse	10	Incristine Sulfate Liposome	20	12	10		
Horton 2019^16^	COG AALL07P1 Study	Children	First relapse	22	Bortezomib + Chemo	68	202		43‐75(3y)(CR2)	43‐75(3y)(CR2)
Xue 2013^21^	Soochow University	Children	Refractory/relapse	11	CAG regimen	90.9	0	9.1	12(1y)	12(1y)
Our Study 2019	Multi‐Centre of China	Adult	Refractory/relapse	41	CAG regimen	80.5	0	66	56.5(2y)	68.8(2y)

Good safety is another advantage of the CAG regimen for R/R‐T‐ALL according to its low hematological or nonhematological adverse events, which is in accordance with previous reports not only in T‐ALL but also in AML.^19^, ^20^, ^21^, ^22^ FLAG and hyper‐CVAD have early death rates of 5%‐20% and 3%‐15%, respectively, and infection rates of 20% to 60%.^17^, ^28^, ^29^, ^30^, ^31^ Infections of grade 3‐4 occurred in 58% of patients during treatment with the CAG regimen in our study, which was similar to other regimens.^17^, ^28^, ^29^, ^30^, ^31^ However, no early deaths (within 1 month) occurred in our study. Novel treatments, such as nelarabine and bortezomib, have the adverse event of neuropathy at a rate of up to 37%, which limits their repetitive use.^8^, ^10^, ^12^, ^16^ However, no patients experienced neuropathy during treatment with the CAG regimen. The favorable safety of the CAG regimen is due to the low doses of araC and aclarubicin plus the use of G‐CSF without using steroids. Previous studies have demonstrated that G‐CSF has the potential to promote the recovery of neutrophils and an enhanced ability to kill phagocytized bacteria,^32^, ^33^ which may partially explain the safety of the CAG regimen.

A high percentage (66%) of patients successfully bridged to allo‐HSCT, translating into promising survival outcomes in our study, while previous studies have shown only 9.1% to 26.9% of patients bridging to allo‐HSCT (Table 3).^7^, ^8^, ^9^, ^10^, ^11^, ^12^, ^13^, ^14^, ^15^, ^16^, ^17^, ^18^ The higher CR rate and lower toxicity of the CAG regimen allow more patients to rapidly proceed to HSCT; this is especially true for R/R‐ETP‐ALL patients, who would otherwise die within 6 months.^23^, ^24^ As shown in our study, of the 26 R/R‐ETP‐ALL patients, 14 successfully bridged to allo‐HSCT after achieving CR, which contributed to the similarly promising outcomes of these ETP‐ALL patients compared with those of the non‐ETP‐ALL patients.

There were some limitations in our study. First, this was a one‐arm trial rather than a randomized study, although we provided a mini‐review of the historical literature. Given the rarity of R/R‐T‐ALL, it is unlikely that large randomized studies in adult patients will be undertaken to examine the question of which regimen is best for salvage therapy. We have initiated a prospective multicenter trial that will include 18 centers in China to validate this study. Second, the mechanism by which the CAG regimen affects T‐ALL cells remains unclear, although a previous study found that T‐ALL cells highly express G‐CSF receptors, and G‐CSF has a synergetic effect in eliminating T‐ALL cells when administered simultaneously with araC and aclarubicin.^34^ Moreover, 24 of 33 refractory patients in our study received only one prior induction regimen, which may introduce a bias and make it difficult to extend our findings to heavily treated patients.

In summary, our multicenter results show that the CAG regimen is highly effective and well‐tolerated in adult patients with R/R‐T‐ALL. The CAG regimen enabled most patients to successfully bridge to allo‐HSCT and achieve improved outcomes; thus, it represents a novel option for this population with poor prognosis.

## CONFLICT OF INTEREST

We declare no competing interests.

## AUTHOR CONTRIBUTORS

H‐HZ, JH, and JJ drafted the manuscript and contributed to the final draft; and others collected data of the manuscript; and all authors reviewed and approved the final draft.

5

**TABLE 1:** The baseline of the patients and their outcomes

Characteristic	Value
Age, y	31(15‐78)
Male sex ‐no. (%)	28(68)
Pretreatment	
White blood cell count (×10^9^/L)	18.8 (0.9‐310.8)
Platelet count (×10^9^/L)	95 (7‐295)
Blasts of bone marrow (%)	81 (21‐99)
Immunophenotyping	
ETP	26(63.4)
Early non‐ETP	6(14.6)
Thymic	6(14.6)
Mature	3(7.3）
Cytogenetics‐no (%)	
Normal	24(58.5)
Complex	6(14.6)
Others	11 (26.8）
Risk	163 (60‐429)
Standard‐risk	14 (34.2)
High‐risk	27 (65.8)
Status before CAG regimen	
Refractory	31
1 Prior course	22
2 Prior courses	6
3 Prior courses	3
Relapsed	10
1 Prior salvage	2
2 Prior salvage	1
No prior salvage	7
Status after CAG regimen	
CR‐no (%)	33 (80.5)
PR‐no (%)	2 (4.9)
NR‐no (%)	6 (14.6)
ORR‐no (%)	35 (84.9)
MRD‐<0.1% after CAG regimen	12 (29.2)
MRD‐>0.1% after CAG regimen	29 (70.8)

Note

High risk was defined as WBC counts higher than 100 × 10^9^/L or complex karyotype at the time of newly diagnosis and others was defined as standard risk. ORR was defined as CR plus PR.

Abbreviations: ETP, early T‐cell precursors; MRD, minimal residual disease detected by flow cytometry.

**TABLE 2:** Incidence of hematological and nonhematological toxic effects during CAG reinduction treatment

Adverse Event	No. of patients				All grades	
	Grade 1	Grade 2	Grade 3	Grade 4	No. of patients	%
Hematologic						
Neutropenia	2	7	4	26	39	95
Anemia	1	8	9	21	41	100
Thrombocytopenia	1	4	2	30	37	90
Nonhematologic						
Infection	1	2	23	1	27	66
Nausea	17	3	0	0	20	49
Vomiting	10	10	0	0	20	49
Hemorrhage	13	1	0	0	14	34
Cardiac	12	1	0	0	13	32
Mucositis	9	2	1	0	12	29
Raised liver ALT/AST	5	1	1	0	7	17
Thrombosis/embolism	6	0	0	0	6	15
Diarrhea	2	1	0	0	3	7
Headache	1	1	0	0	2	5
Hyperbilirubinemia	2	0	0	0	2	5
Rash	1	0	0	0	1	2
Raised creatinine	0	0	0	0	0	0

Abbreviation: ALT, alanine transaminase; AST,aspartate aminotransferase.
